# Low-Temperature Gas-Phase Kinetics of Ethanol–Methanol Heterodimer Formation

**DOI:** 10.1021/acs.jpca.3c01312

**Published:** 2023-04-29

**Authors:** Lincoln Satterthwaite, Greta Koumarianou, P. Brandon Carroll, Robert J. Sedlik, Irene Wang, Michael C. McCarthy, David Patterson

**Affiliations:** † Department of Chemistry and Biochemistry, Building 232, University of California, Santa Barbara, California 93106, United States; ‡ Center for Astrophysics | Harvard & Smithsonian, 60 Garden Street, Cambridge, Massachusetts 02138, United States; ¶ Physics Department, Broida Hall, University of California, Santa Barbara, California 93106, United States

## Abstract

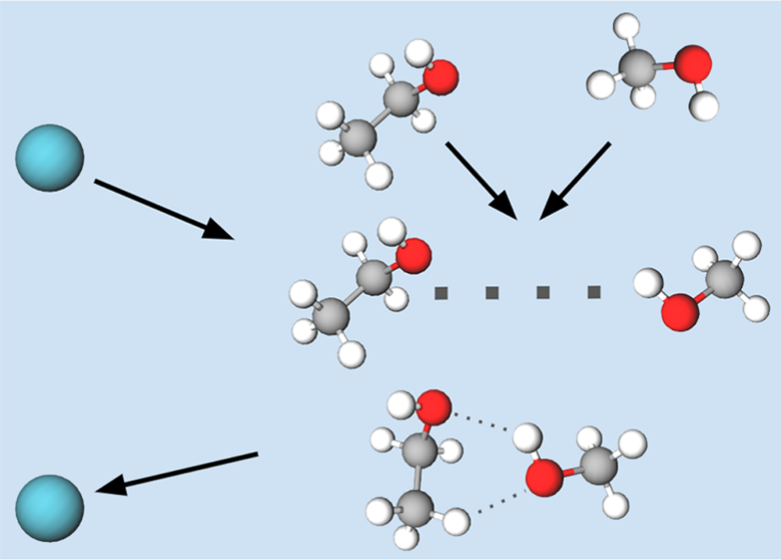

The structures of gas-phase noncovalently bound clusters have long been studied in supersonic expansions. This method of study, while providing a wealth of information about the nature of noncovalent bonds, precludes observation of the formation of the cluster, as the clusters form just after the orifice of the pulsed valve. Here, we directly observe formation of ethanol–methanol dimers via microwave spectroscopy in a controlled cryogenic environment. Time profiles of the concentration of reagents in the cell yielded gas-phase reaction rate constants of *k*_*Me*-*g*_ = (2.8 ± 1.4) × 10^–13^ cm^3^ molecule^–1^ s^–1^ and *k*_*Me*-*t*_ = (1.6 ± 0.8) × 10^–13^ cm^3^ molecule^–1^ s^–1^ for the pseudo-second-order ethanol–methanol dimerization reaction at 8 K. The relaxation cross section between the gauche and trans conformers of ethanol was also measured using the same technique. In addition, thermodynamic relaxation between conformers of ethanol over time allowed for selection of conformer stoichiometry in the ethanol–methanol dimerization reaction, but no change in the ratio of dimer conformers was observed with changing ethanol monomer stoichiometry.

## Introduction

Gas-phase noncovalently bound clusters of molecules form an important class of structures in chemistry. Gas-phase molecule aggregation plays an important role in a variety of processes, including astrochemistry^1−5^ and atmospheric chemistry.^6−9^ While the structure of noncovalently bound clusters has been studied with many techniques,^10−14^ observation of the gas-phase process monomer + monomer → dimer has not been directly observed for a heterodimer.

Techniques like ultrafast infrared spectroscopy can easily resolve the time scales of gas-phase molecule aggregation but are disadvantaged in studying this type of diffuse gas-phase system because of the small interaction region of a typical laser driven pump–probe experiment.^15^ Gas electron diffraction (GED) has powerful applications in intramolecular dynamics and structure determination in gas-phase samples, and in many cases provides a useful supplement to microwave spectroscopy in structure elucidation.^16−18^ The application of GED to studying cluster formation dynamics is challenging, as the confusion limit and signal-to-noise of GED is low, and clusters formed in situ typically have a density on the order of 1% of the parent species value.^19^

The workhorse technique for experimentally studying the structure of small clusters is microwave spectroscopy, typically carried out in a pulsed seeded supersonic expansion.^20−24^ High valve backing pressures yield high three-body collision rates and thus efficient cluster formation in the volume immediately downstream of the valve orifice. This volume has a strong density gradient and rapidly changing temperatures, which precludes precise control over formation conditions. The interaction region of seeded supersonic expansion experiments is typically far downstream of the pulsed valve, only allowing for study of already formed clusters. While a buffer gas cell has much lower cluster formation efficiency, the clusters form under controlled conditions in the interaction region of the experiment, enabling the possibility of direct observation by microwave spectroscopy.

CRESU (Cinétique de Réaction en Ecoulement Supersonique Uniforme - French acronym for uniform supersonic flow) flows have been used extensively to study the reaction dynamics of highly reactive charged species, as well as neutral open and closed shell molecules. This technique uses a pulsed or continuous expansion from a high pressure backing chamber through a Laval nozzle, creating a cold, uniform, high flux beam of molecules entrained in a carrier gas. Rotational and vibrational degrees of freedom are efficiently cooled, with supersonic lab-frame flow velocities. Three-body collisions in the beam allow for the formation of weakly bound complexes, like water clusters, as well as association reactions like methanol and CN to form acetonitrile.^25,26^ The addition of chirped pulse Fourier transform microwave (CP-FTMW) instruments to CRESU to perform chirped pulse uniform flow (CPUF)^27^ can enable isomer, conformer, and site-specific isotopologue monitoring of reactants and products.

However, CPUF systems have several restrictions that limit their applicability to some chemical systems. Uniform supersonic flows have a base temperature of about 20 K and require high flows of carrier gas and molecular sample to maintain the expansion with appropriate concentration, typically of order 3 × 10^3^ m^3^ h^–1^ of carrier gas and <1% sample.^28^ Such high sample consumption precludes the use of high cost samples and almost all isotopically enriched samples. Furthermore, due to the hydrodynamic time scales of the supersonic flow, observations are limited to 1 ms or less. Finally, large pressure broadening, the use of skimmers, and room temperature low noise amplifiers limits the absolute sensitivity achievable in CPUF experiments. Practically, this limits the measurable rate constants and precludes the observation of minor products, isotopic labeling, and measurement of kinetic isotope effects.

Here, we present the conformer-selected formation of ethanol–methanol dimers in a cryogenic buffer gas cell and provide rate constants and collisional cross sections for these reactions. Many organic molecules have several conformers present at room temperature, and these conformers tend to relax to the lowest energy conformer in cryogenic buffer gas environments.^29^ By waiting for several thousand collisions with cold helium over several tens of milliseconds, the ground-state conformer is enriched. Introduction of the other component of the dimer, in this case methanol, can be delayed until the sample is well-thermalized and the ground-state conformer dominates. In this way, the dimerization reaction can be selected to occur with the lower energy trans conformer of ethanol in higher stoichiometry.

Prior to this study, it was unclear whether the energy of a dimer-forming collision would “scramble” the conformation of the monomer in resulting dimer. By observing the ratio of amplitudes of a few species-specific rotational transitions in the well-documented methanol–trans-ethanol (ME-T) and methanol–gauche-ethanol (ME-G) dimers,^30^ the relative abundance of these dimers as a function of ground-state ethanol abundance was measured for the first time, showing that the formed dimer has no memory of the conformation of the monomer that participated in the reaction.

## Methods

Two different buffer gas cells were used to take these measurements; the major difference being the size of the buffer gas cell. Kinetic data were taken at UCSB, and both the CfA and UCSB buffer gas cells were used to measure relaxation rates and collisional cross sections. Using two similar experiments with fairly different configurations was important to verify that we were, in fact, measuring dynamics of buffer gas and molecule interactions as opposed to measuring some characteristic of the buffer gas cell in which the measurement was conducted.

**Figure 1:**
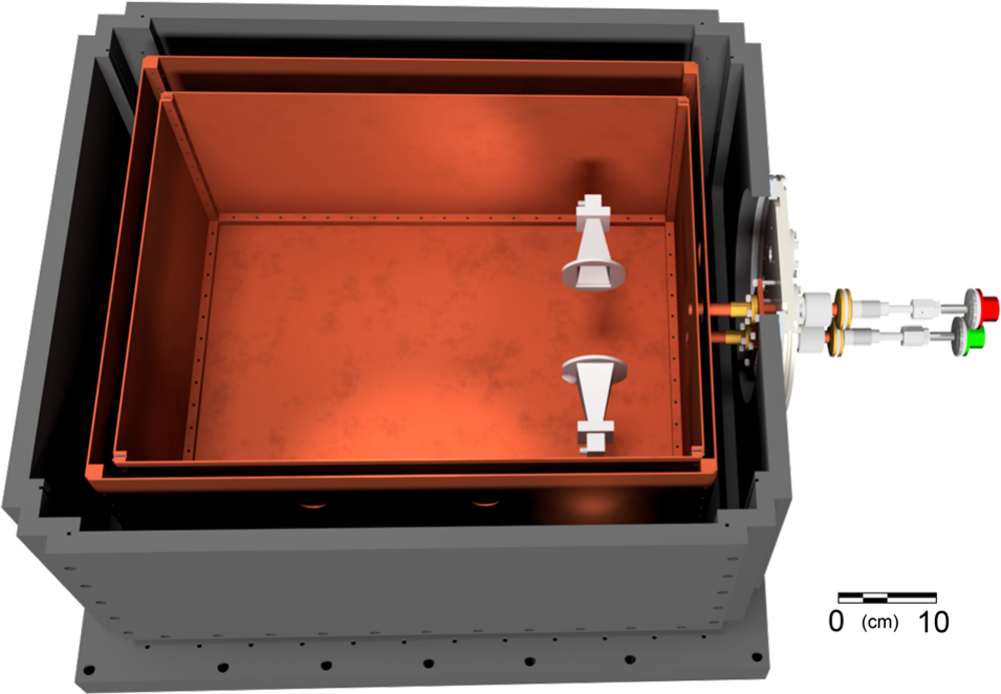
UCSB experimental apparatus. Valve 1 (red) was backed by ethanol, and valve 2 (green) was backed by methanol each at their respective room temperature vapor pressures. Horns were located to provide the best overlap with the expanding gas cloud. The buffer gas cell is roughly 45 cm × 25 cm × 27 cm.

The apparatus used at UCSB is shown in Figure 1, and the electronics are described in ref (31). Helium was continuously flowed at 10 standard cubic centimeters per second (SCCM), resulting in a steady-state helium density of roughly *n*_*He*_ =  cm^–3^, where *f* is flow in molecules per second, *A* is total area of all apertures in the cell, and *v*_*He*_ is the thermal velocity of helium at 8 K.

At standard temperature and pressure conditions, dimers of small hydrogen bonding molecules are present at the 0.1% level,^32^ a concentration easily visible in this experiment. To enforce separation of the molecular samples until they react within the interaction region of the apparatus, two separate but identical sample input manifolds were employed in experiments performed at UCSB (Figure 1).

**Figure 2:**
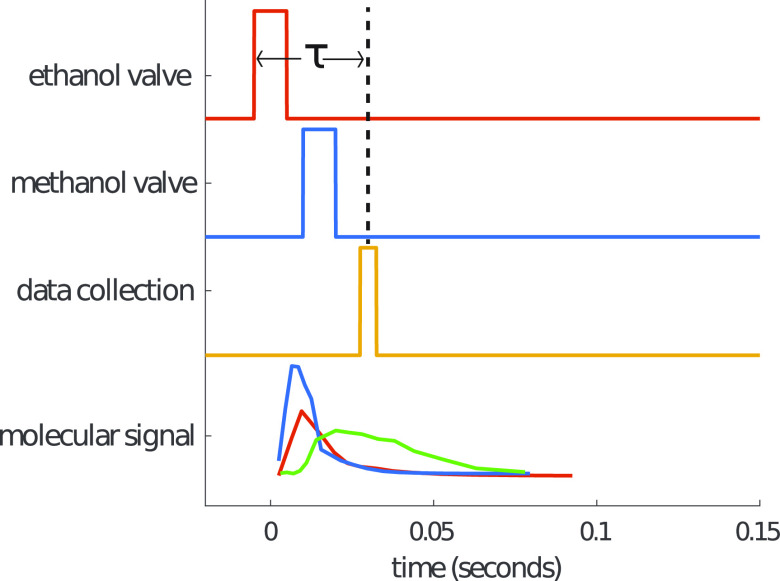
Timing diagram for the experiment. The delay between the first valve and the center of the data collection window is τ. The delay between the ethanol valve and methanol valve is adjusted to change the effective stoichiometry of the dimer-forming reaction. The colors on the bottom trace correspond to the timing trace of the same color, with the green trace being the dimer signal.

Each sample input manifold was equipped with a Parker series 9 pulsed valve operating at 0.8 Hz, backed by either ethanol or methanol at room temperature vapor pressure, 48 and 102 Torr, respectively, and no carrier gas. This repetition rate was selected to optimize data acquisition speed while maintaining low buffer gas temperatures. The delay between these valves was set to values between 0 and 40 ms, with ethanol leading for the conformational memory study. The low backing pressures resulted in very little cooling by expansion after each valve shot; the pulsed valves are only used to gate the experimental cycle as shown in Figure 2.

The apparatus used at the Center for Astrophysics (CfA) is described in ref (33) with electronics largely unchanged from that reference. Different aperture sizes on this apparatus resulted in a steady-state buffer gas density of *n*_*He*_ = 3.2 × 10^14^ cm^–3^ at buffer gas flows of 10 SCCM. On the CfA apparatus, only one pulsed valve was used for ethanol, backed by room temperature vapor pressure, and methanol was continuously flowed through a different input into the cell at 0.1 SCCM. The ethanol-backed pulsed valve was opened for 1 ms at 5 Hz.

Signal was collected by polarizing species-specific rotational transitions and recording the resulting rotational free induction decays, as in a typical Fourier transform microwave spectroscopy experiment. Data collection was started some time delay τ – (*T*_*window*_/2) after the valve opening time and halted at time τ + (*T*_*window*_/2), effectively strobing data collection (Figure 2). This approach allows for a time-domain picture of signal amplitudes for each species in the buffer gas cell at a given time to be acquired, with the constraint that there must be a transition within the chirp bandwidth for each species to be observed.

To derive rate constants from the data taken in this experiment, there must be a calibration between the raw signal amplitude and the density of molecules in the cell at a given time. To perform this calibration, a direct microwave absorption measurement was taken on methanol, which has a well-known dipole moment,^34^ and thus with a straightforward calculation was converted to molecular density. This procedure yielded in a peak density of 2 ± 0.5 × 10^12^ cm^–3^ for methanol, and about 10^9^ cm^–3^ for all dimer species. This value for peak density was then combined with the linear fit for the rise time of the dimer signal, resulting in a gas-phase rate constant for each of these dimerization reactions.

The value for density derived from absorption was checked by measuring the valve reservoir depletion over time. The pulsed valve was backed with a 20 μL sample of methanol, and the depletion of the reservoir as a function of number of valve pulses was measured by plotting signal amplitude over an hour of runtime, or 2880 valve pulses. From the known quantity of methanol backing the valve and number of valve pulses, a number of methanol molecules per pulse was extracted. The ratio of the number of molecules per pulse to the volume of the buffer gas cell yields a ceiling of molecular density in the cell, 2.3 ± 1 × 10^12^ cm^–3^, which agrees well with the absorption measurement.

## Results and Discussion

### Diffusion and Relaxation Cross Section of Ethanol

Ethanol has long been studied in supersonic expansion experiments, with thermodynamic population ratios of its gauche and trans states. Trans is the ground state, followed by the quasi-degenerate gauche+ and gauche– conformers 56 and 61 K above the ground state, respectively.^35,36^ Relaxation between these two states, however, has not yet been directly observed. In experiments performed in a supersonic expansion, a room-temperature thermodynamic distribution of ethanol conformers persisted when seeded with helium, but not with a heavier carrier gas, in stark contrast to the results presented here.^37^ A possible explanation is that, in our system, the buffer gas is already cold, and the molecules come in at high temperature, so the initial average collision energy is high. In a supersonic expansion, where the molecular sample and carrier gas cool simultaneously, the average collision energy after the initial expansion is quite low, so a heavier collision partner is necessary for conformational relaxation to occur. Extensive theoretical calculations of this relaxation process have been performed for many molecules, but this is one of only a few molecules to be directly observed to relax to a lower energy conformer due to cold collisions.^29,38−40^

Short pulses of ethanol were introduced to the cell as described above, and diffusion through the buffer gas to the walls over an effective cell area was measured by fitting the free induction decay (FID) of the molecule to a decaying exponential. Following the method of Drayna and co-workers,^29^ a diffusion model was fit to the data. The differential equation
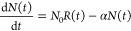
1where *N*_0_ is the total number of molecules introduced per pulse, *R*(*t*) is sample introduction rate as a function of time,  where τ is the zero-order diffusion time, and *N*(*t*) is the number of molecules in the interaction region of the experiment as a function of time describes the system.

The loading rate
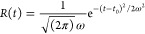
2where ω and *t*_0_ are properties of the pulsed valve, describes a Gaussian pulse shape from the pulsed valve in the sample introduction manifold. The solution to differential eq 1 is
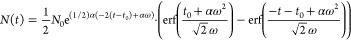
3

**Figure 3:**
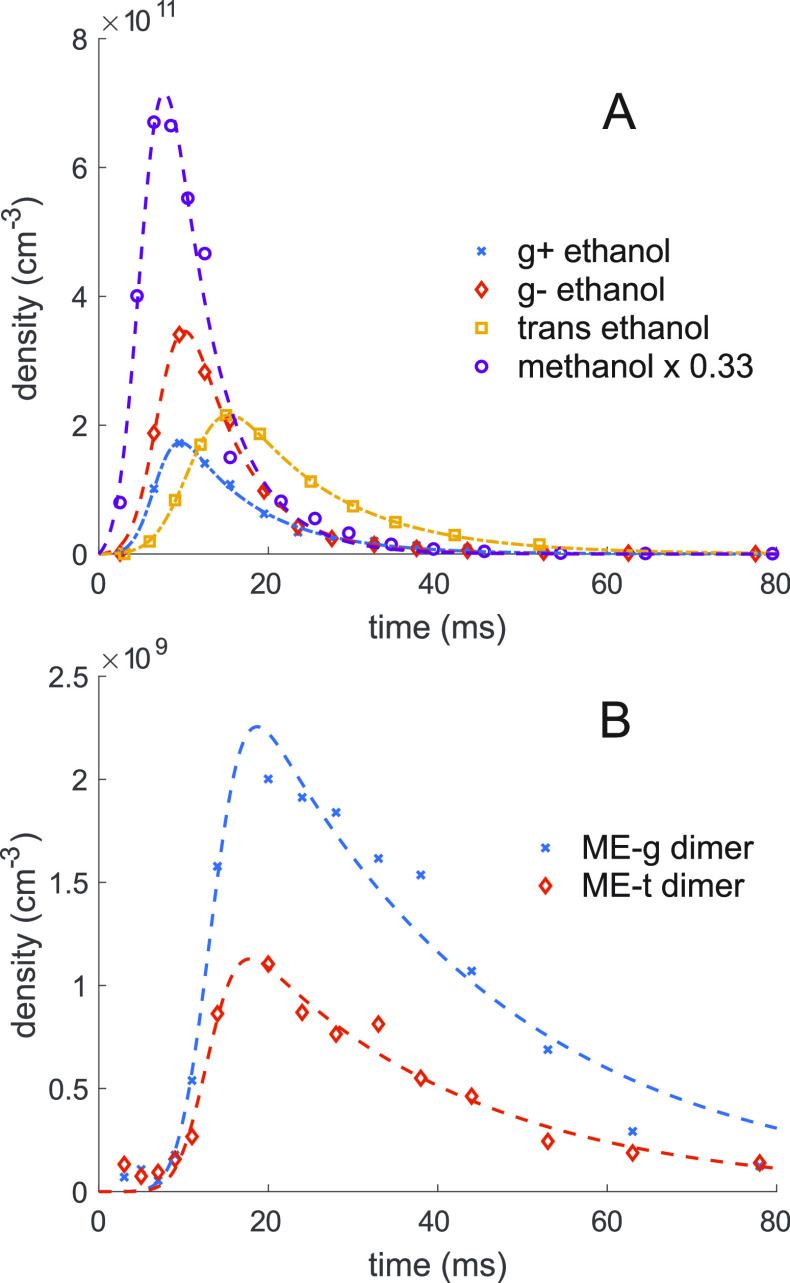
Time profile of relative populations of (A) all monomers and (B) the two lowest energy dimer conformations. A sample introduction and diffusion model, eq 3, was fit to the data. Fits from the rising side give reaction rate constant, and fits from the falling side give diffusion rates inside the buffer gas cell. Fit parameters for diffusion are shown in Table 1.

The decay time τ_*FID*_ is equal to the mean time between collisions, and by combining the mean free time of the molecular sample with the diffusion time, the relaxation cross section of 2.3 × 10^–17^ cm^2^ is extracted for ethanol. This is 3 orders of magnitude smaller than the elastic collisional cross section, suggesting that conformational relaxation requires a few hundred collisions with cold buffer gas. The gauche+ and gauche– species of ethanol are nearly degenerate, separated by only 5 K,^41^ so the amplitude of identical transitions in each species was summed and treated as a single species. The diffusion cross sections were calculated following the approach of Hutzler and co-workers.^42^ The expression
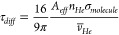
4derived from Chapman–Enskog theory, describes the relation between diffusion time τ_*diff*_, from the model fit to the data in Figure 3, effective cell area *A*_*eff*_, buffer gas velocity *v̅*_*He*_ and density *n*_*He*_, and diffusion cross section σ_*molecule*_.

The diffusion cross sections of trans and gauche ethanol are within 1%, so the diffusion times of each ethanol species, omitting other loss pathways, are also essentially identical. Other loss pathways for ethanol include dimerization, either with another ethanol molecule or with a methanol molecule, and with the exception of the ground-state trans monomer, relaxation to a lower energy conformer. Of these nondiffusive loss pathways, relaxation is by far the dominant loss mechanism for gauche ethanol.

### Dimer Formation Rates

Absorption on the strongest methanol line in our frequency range, the |2_0_⟩–|3_–1_⟩ transition at 12178.58 MHz, was measured to be 1.2% in the UCSB apparatus. With a photon absorption cross section of
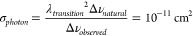
5the Beer–Lambert expression *A* = *nlσ*_*photon*_ is applied. This result is then multiplied by a correction factor of 1 × 10^3^ to account for fractional-state occupation at a rotational temperature of 8 K, yielding an in-cell density of 2 × 10^12^ cm^–3^. This was used to associate a measured voltage out of the experiment in regular chirped-pulse mode with an in-cell density in order to derive rate constants based on signal rise time. Measured voltage out of the experiment is linearly proportional to the magnitude of μ_*a*_, μ_*b*_, or μ_*c*_, for an A-type, B-type, or C-type transition, respectively, for the case of optimized drive pulse strength (strong,  pulse limit).^43^ Measured voltage out is also linearly proportional to in-cell molecular density, so by combining these two known quantities, densities for other monomers and dimers which lack the required density for a direct absorption measurement can be estimated. Peak dimer concentration was calculated to be 2.5 × 10^9^ cm^–3^ for ME-g and 1.1 × 10^9^ cm^–3^ for ME-t by this approach.

In Figure 3, the temporal profile of the rising side of the curve, nonzero points at time less than 15 ms, were linearly fit, with the slope of this curve giving  in the rate equation
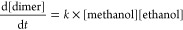
where *k* is the rate constant and brackets around reagents indicate concentration in units of cm^–3^. Interpolating from the absorption measurement above, the concentration of ethanol was 3.5 × 10^11^ cm^–3^, based on μ_*a*_ dipole moment of 1.264 D and a fractional-state occupation of 0.014 measured at 8 K at the |1_01_⟩–|0_00_⟩ transition at 17288.41 MHz. Combining these three quantities, a rate constant *k*_*Me-g*_ = (2.8 ± 1.4) × 10^–13^ cm^3^ molecule^–1^ s^–1^ for the formation of ME-g and *k*_*ME*-*t*_ = (1.6 ± 0.8) × 10^–13^ cm^3^ molecule^–1^ s^–1^ for ME-t was measured.

### Conformer-Selected Dimer Formation

**Figure 4:**
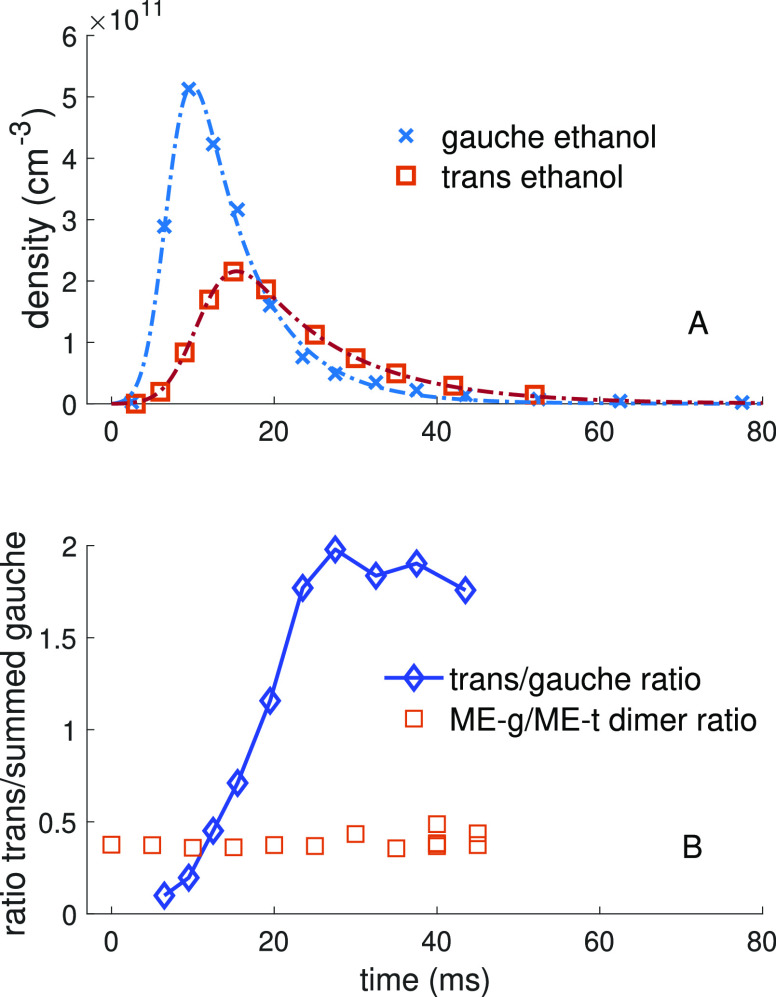
(A) Time-domain plot of reactants. The gauche ethanol trace represents the sum of g+ and g– conformer densities, as these conformers are quasi degenerate. Data are fit to the theoretical diffusion model described in the Results and Discussion. (B) As the ratio of gauche and trans ethanol monomer changes, the ratio ME-g and ME-t does not change.

The highest energy (gauche+) and lowest energy (trans) of three possible conformations of ethanol are separated by only 64 K.^44^ In a room temperature sample, this means that there is a roughly equal fraction of all three conformers, but after thermalization with a cryogenic buffer gas, the higher energy conformers relax to the ground-state trans conformer with a relaxation cross section of 2.3 × 10^–17^ cm^2^, derived from diffusion times measured by fitting exponential decays to monomer populations over time (Table 1 and Figure 4A). As relaxation proceeds over time, the fraction of ethanol in the gauche conformer is reduced, and methanol can be introduced at varying times to expose it to different varying stoichiometries of ethanol monomers. The ratio of the amplitudes of the 16655.448 MHz transition of ME-g and the 14266.636 MHz transition of ME-T plotted against the fraction of ethanol in the trans conformation reveals any memory of the conformation of the monomer in the resulting dimer and can be seen in Figure 4B.

**Table 1:** Table of Fit Parameters and Derived Quantities for the Exponentially Decaying Parts of the Dimer Movies^a^

quantity	symbol	UCSB value	CfA value	unit
τ*_decay_*	τ_*D*,*MeOH*_	6.5		ms
	τ_*D*,*t*-EtOH_	14.1	2.68	ms
	τ_*D*,*g*-EtOH_	10.0	1.06	ms
	τ_*D*,*ME*-*g*_	26.7	4.95	ms
	τ_*D*,*ME*-*t*_	30.7	4.42	ms
diffusion cross section	σ_*D,MeOH*_	1.1 × 10^–14^		cm^2^
	σ_*D,t-EtOH*_	2.2 × 10^–14^	2.1 × 10^–14^	cm^2^
	σ_*D,g-EtOH*_	1.4 × 10^–14^	1.1 × 10^–14^	cm^2^
	σ_*D*,*dimer*_	4.9 × 10^–14^	3.5 × 10^–14^	cm^2^
relaxation cross section	σ_*c*,*gt*_	2.3 × 10^–17^	3.3 × 10^–17^	cm^2^
reaction rate constant	*k*_*ME*-*g*_	2.8 ± 1.4 × 10^–13^		cm^3^ molecule^–1^ s^–1^
reaction rate constant	*k*_*ME-*–*t*_	1.6 ± 0.8 × 10^–13^		cm^3^ molecule^–1^ s^–1^

a τ is extracted from the downslopes of movie traces, and the collisional cross section is calculated as described in Diffusion and Relaxation Cross Section of Ethanol.

The lack of change in the ratios of the amplitude of dimer transitions versus valve delay suggests that each collision between monomers effectively “scrambles” the conformational landscape of the monomer, and the resulting conformation of the dimer is quasi-random. There was no conformational relaxation observed in the dimer species on the time scale of this experiment.

## Conclusions

We present a new technique for determining relatively slow gas-phase reaction rate constants, compatible with expensive or rare samples. Cryogenic gas-phase rates of formation for hydrogen bonded clusters of methanol–ethanol were determined, with conformer specificity and the relaxation cross section for ethanol around 8 K was also determined. The dependence of ultimate conformation of the hydrogen bonded cluster on the conformation of the ethanol monomer was also investigated, and we report a null result. The case presented here where the energy of a collision is just enough to “scramble” the conformational landscape of the reaction partner does not present the whole story, as ethanol is somewhat unusual in the very close energies of its conformers. Other species with larger energetic differences between conformers may not exhibit the same behavior in their dimerization reactions and should be further investigated.
